# The knowledge, attitude, and practice score on oral nutrition supplementation is inversely associated with malnutrition risk of tumor patients during the peri-radiotherapy period: a multicenter cross-sectional study

**DOI:** 10.3389/fnut.2026.1754291

**Published:** 2026-04-29

**Authors:** Shasha Zhao, Cong Wang, Henan Zhang, Shengsi Yang, Qin Du, Haijun Lu, Liang Cao, Haitao Wan, Yali Miao, Bo Li, Baorong Xiao, Yanxing Sheng, Shucheng Ye, Ming Lu, Shanghui Guan

**Affiliations:** 1 Department of Clinical Nutrition, Qilu Hospital of Shandong University, Jinan, China; 2 Department of Radiation Oncology, Shanghai Pulmonary Hospital, Tongji University School of Medicine, Shanghai, China; 3 Department of Radiation Oncology, Qilu Hospital of Shandong University, Cheeloo College of Medicine, Shandong University, Jinan, China; 4 Abbott Laboratories, Shanghai, China; 5 Department of Radiotherapy, Affiliated Hospital of Qingdao University, Qingdao, China; 6 Department of Radiotherapy, Taian Tumor Prevention and Treatment Hospital, Taian, China; 7 Department of Radiotherapy, Qingdao Central Hospital, Qingdao, China; 8 Department of Radiotherapy, The First People's Hospital of Jining, Jining, China; 9 Department of Radiotherapy, Yantai Yuhuangding Hospital, Yantai, China; 10 Department of Radiotherapy, Taian Central Hospital, Taian, China; 11 Department of Radiotherapy, Liaocheng People's Hospital, Liaocheng, China; 12 Department of Radiotherapy, Affiliated Hospital of Jining Medical University, Jining, China; 13 Department of Thoracic Surgery, Shanghai Pulmonary Hospital, Tongji University School of Medicine, Shanghai, China

**Keywords:** attitudes, knowledge, malnutrition risk, oral nutrition supplementation, peri-radiotherapy period, practices

## Abstract

**Background:**

Oral nutritional supplements (ONS) plays a key role in improving outcomes in cancer patients undergoing radiotherapy. However, the relationship between patients’ knowledge, attitude, and practice (KAP) toward ONS and malnutrition risk remains unclear.

**Methods:**

This multicenter cross-sectional study included 1737 cancer patients from 25 hospitals in Shandong, China. KAP toward ONS was assessed using a structured questionnaire. Logistic regression analyses were performed to evaluate the association between KAP scores and malnutrition risk.

**Results:**

The prevalence of malnutrition risk was 70.1%. Higher KAP scores were independently associated with lower malnutrition risk. Patients in the highest KAP quartile had a significantly reduced risk compared with the lowest quartile (adjusted OR = 0.48, 95% CI: 0.35–0.66). Education level was positively associated with KAP scores.

**Conclusion:**

Higher KAP toward ONS is significantly associated with reduced malnutrition risk in cancer patients during the peri-radiotherapy period. Improving patient education and standardized nutritional management may represent effective strategies for reducing malnutrition risk in clinical practice.

**Limitation:**

Due to the cross-sectional design, causal relationships cannot be established.

**Clinical trial registration:**

Identifier ChiCTR1800019983.

## Background

Malnutrition has adverse effects on the treatment and prognosis of patients undergoing radiotherapy (1–3). The global incidence of malnutrition in cancer patients ranges from 20% to more than 70%, being most prevalent in those with gastrointestinal, head and neck, liver, and lung cancers (4). In a cohort study, 70.3% of patients were identified as malnutrition using the Global Leadership Initiative on Malnutrition (GLIM) criteria in China (5). The consequences of malnutrition in cancer patients include weight and muscle loss (6), reduced immunity and susceptibility to infection (7, 8), psychosocial stress (9), poor quality of life (10), therapeutic toxicity (11), greater risk of death (12, 13), reduced survival, and increased medical costs (14). In addition, 10–20% of cancer patients’ deaths can be attributed to malnutrition rather than malignant tumors themselves (12, 15). Therefore, nutritional screening and nutritional therapy are important parts of comprehensive cancer management.

A nutritional intervention aims to maintain or improve food intake, alleviate metabolic disorders, maintain skeletal muscle quality and body function, reduce or interrupt the risk of scheduled anticancer therapy, and improve quality of life (16). Effective nutritional intervention for cancer patients can reduce hospitalization expenses, follow-up loss during treatment, postoperative infection, and hospitalization time by improving the nutritional status (17). The available guidelines recommend that the standard treatment of malnutrition should follow the five-step therapy principle, in which oral nutrition supplements (ONS) is the preferred medical nutrition route besides nutrition education (18–22).

Still, few studies on the nutritional status and nutritional intervention status of the patients before, during, and after radiotherapy are available, especially regarding the nutritional intervention and nutritional status during the whole peri-radiotherapy period. In addition, although the importance of nutrition in oncology patients is well established, malnutrition remains frequently overlooked in the clinic. Understanding the patients’ knowledge, attitude, and practice (KAP) towards malnutrition and nutrition therapies might help understand the high malnutrition risk.

Despite increasing awareness of cancer-related malnutrition, evidence on patients’ knowledge, attitudes, and practices toward ONS across the entire peri-radiotherapy period remains limited. This study addresses this gap by evaluating KAP toward ONS in a large multicenter cohort and examining its association with malnutrition risk. We hypothesized that higher KAP scores would be independently associated with a lower risk of malnutrition throughout the peri-radiotherapy period.

## Methods

### Study design and participants

This study was a multicenter cross-sectional survey of hospitals in Shandong Province, performed from November 2018 to February 2019. The 25 hospitals that participated in the survey included the level III and level II hospitals for sampling convenience. The eligibility criteria for the patients were (1) ≥ 18 years of age, (2) scheduled for radiotherapy within 2 weeks, receiving radiotherapy, or returning for follow-up within 3 months after radiotherapy, (3) mentally clear and able to independently comprehend and complete the questionnaire independently; and (4) willing to participate and signed the informed consent form. The patients were surveyed either in the radiotherapy ward, the waiting room of the radiotherapy department, or the waiting room of the outpatient clinic. The physicians were surveyed at their offices. The study protocol was approved by the Ethics Committee of Qilu Hospital, Shandong University, and all participants provided written informed consent.

### Investigation tools

The self-designed questionnaires integrated the published literature (23–28). The questionnaire on the nutrition status of patients in radiotherapy in Shandong province was used to determine the status of patients’ KAP towards ONS before, during, and after radiotherapy (Supplementary File 1). The questionnaire collected patients’ clinical information, including gender, age, tumor site, clinical stage, treatment type, and phase of radiotherapy. The Knowledge section focused on patients’ understanding of ONS and malnutrition information. This section also inquired about their sources of nutritional knowledge. The Attitude section assessed patients’ concerns regarding nutritional improvement during the peri-radiotherapy period. The Practice section evaluated the timing of initiation, specific methods, duration of use, and dosage of ONS during the peri-radiotherapy period.

Each organization was responsible for conducting the survey, with procedures adapted to their local practice and patient flow. For inpatients, it was suggested to provide the survey during the evening shift or just before discharge. For outpatients, it was suggested to be completed when the patient came to the hospital. Each center had a project leader responsible for the organization of the center’s project investigation, including questionnaire quality control, drawing up a list of patients eligible for inclusion, and assigning the tasks to the doctor-in-charge. Ethical approval for the study was obtained prior to study commencement from the Ethics and Scientific Review Committee of Shandong University Qilu Hospital (approval number: QLYY2018-145), and all participants signed written informed consent. Patient privacy was ensured through data anonymization and secure encrypted storage.

### Screening for malnutrition

Nutritional Risk Screening 2002 (NRS2002) was in higher accordance with the Global Leader Initiative on Malnutrition (GLIM) criteria than the Malnutrition Universal Screening Tool (29). The NRS2002 is widely used for nutritional screening in Chinese hospitals. The total nutritional risk score is calculated as the sum of the impaired nutritional status score, the severity of disease score, and an age adjustment. The severity of disease score is associated with the diseases decreasing food intake. The impaired nutritional status score is associated with BMI, body weight and impaired food intake. The total score ≥3 indicates that the patient is nutritionally at risk and a nutritional care plan is initiated (Supplementary File 2).

### Statistical analysis

The prevalence of nutritional risk among cancer patients undergoing radiotherapy ranges from 40 to 80% (30). Considering *α* = 0.05, *β* = 0.10, and a prevalence rate of 40%, at least 600 patients were required. This study was first planned to investigate 2000 patients, indicating that the power would be sufficient to examine the nutrition-related KAP of radiotherapy patients.

Microsoft Access 10.0 was used for data management. The valid questionnaires were entered into the Access database by trained personnel. The difference between the actual number of respondents and the actual number of valid questionnaires was used to reflect on-site quality control. The percentage of patients with malnutrition risk was expressed as the percentage of the number of people with NRS2002 score ≥3 in the actual number of respondents, with the 95% confidence interval (CI) being reported. The categorical variables are described as *n* (%). The continuous variables are described as means and standard deviations or medians and inter-quartile ranges, respectively, according to the normal/skewed distribution. Univariable and multivariable logistic regression analyses were performed. The multivariable model revealed that higher KAP scores were associated with significantly lower malnutrition risk (adjusted OR = 0.48, 95% CI: 0.35–0.66). The significant association between KAP scores and malnutrition risk was further supported by the odds ratio (OR = 0.48, 95% CI: 0.35–0.66), indicating that patients with higher KAP scores were nearly 50% less likely to be at risk of malnutrition. Statistical analyses were performed using IBM SPSS Statistics for Windows, Version 25.0 (IBM Corp., Armonk, NY, USA).

## Results

### Study participants

**Figure 1:**
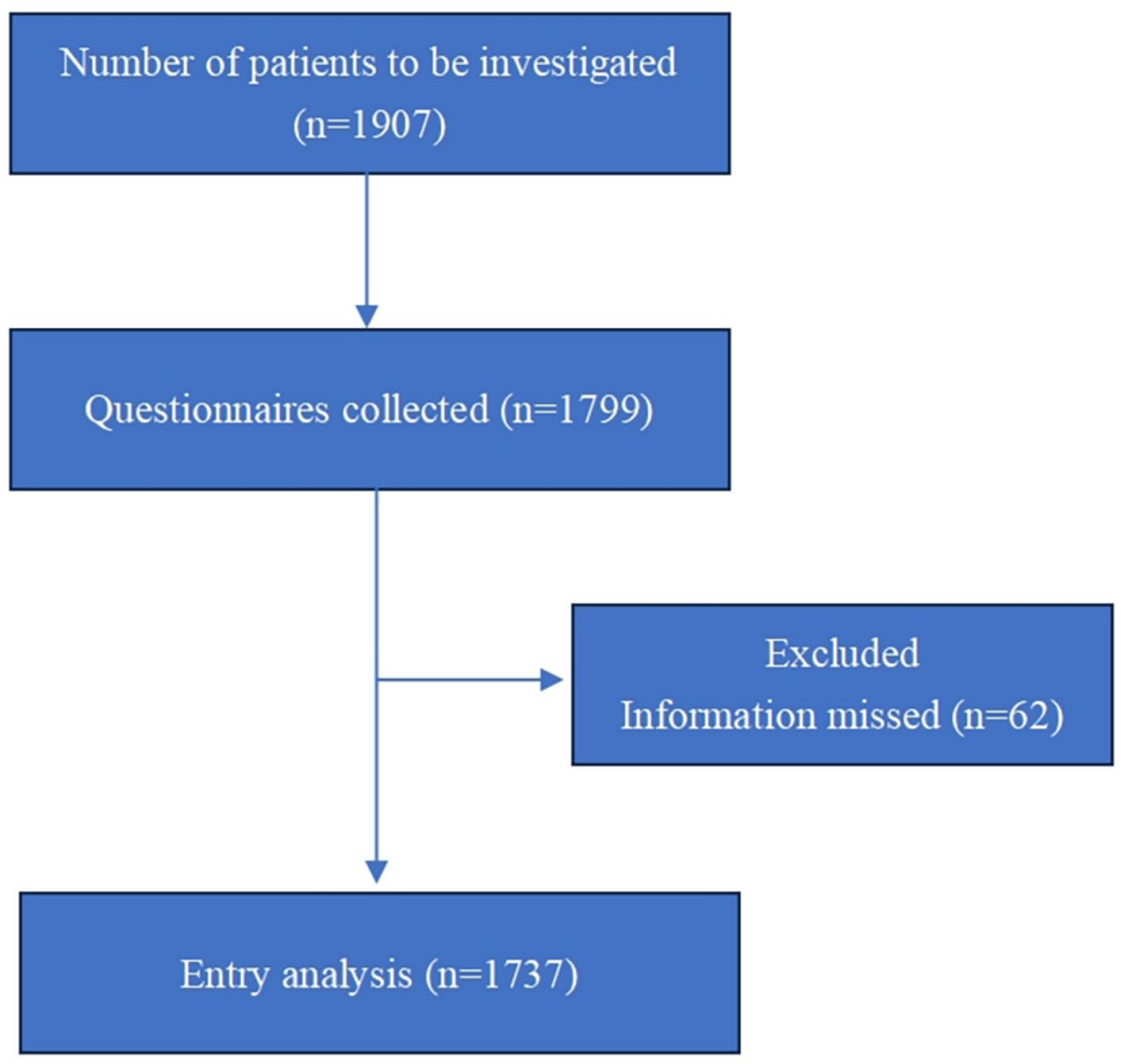
CONSORT diagram depicting flow of participants.

The radiotherapy departments of 25 hospitals in Shandong Province surveyed 1907 patients during the study period, and 1799 valid questionnaires were collected (response rate of 94.3%). After excluding questionnaires with missing information, 1737 patients were included in the final analysis (Figure 1). As shown in Table 1, 54.9% (953/1737) of the subjects were male, and 45.1% (784/1737) were female. Number of patients younger than 70 years old was 1,417(81.6%) and 320(18.4%) was over 70 years old. The mean age of the cohort was 58.94 ± 12.04 years. In terms of patients’ education level, 3.8% (66/1737) of patients did not receive education, 33.6% (584/1737) received informal education, 31.0% (539/1737) received primary education, 21.5% (374/1737) received secondary education and 10.0% (174/1737) received tertiary education. In terms of therapy stage, 13.3% (231/1737) of the respondents were in the preparation stage before radiotherapy, 59.8% (1,038/1737) of the patients were during radiotherapy stage, and 26.9% (468/1737) of the patients were in the 3-month follow-up period after radiotherapy. In terms of clinical stage, 4.7% (81/1737) of patients were stage I and 13.1% (228/1737) were stage II, 25.3% (439/1737) were stage III and 31.8% (553/1737) were stage IV. The treatment modalities included radiotherapy alone (42.4%), concurrent chemoradiotherapy (25.6%), and sequential chemoradiotherapy (17.7%). Patients of thoracic cancer (56.5%), gastrointestinal cancer (14.1%) and head and neck cancer (13.5%) predominated.

**Table 1:** Sociodemographic characteristics of respondents.

Variables	Classification	Frequency (*n* = 1737)	Percentage (%)
Gender	Male	953	54.9
	Female	784	45.1
Age	<70 years	1,417	81.6
	≥70 years	320	18.4
Education level	No education	66	3.8
	Informal education	584	33.6
	Primary education	539	31.0
	Secondary education	374	21.5
	Tertiary education	174	10.0
Radiotherapy stage	Before	231	13.3
	Middle	1,038	59.8
	After	468	26.9
Clinical stage	I	81	4.7
	II	228	13.1
	III	439	25.3
	IV	553	31.8
Therapy	Radiotherapy only	737	42.4
	Concurrent chemoradiotherapy	444	25.6
	Sequential chemoradiotherapy	308	17.7
Cancer type	Head and neck cancer	235	13.5
	Thoracic cancer	982	56.5
	Gastrointestinal cancer	245	14.1
	Others	80	4.6

Nutritional risk screening for malignant tumor patients is the first step in nutritional diagnosis and treatment. The prevalence of malnutrition risk was 70.1% (1,218/1737), indicating a substantial clinical burden. The KAP survey demonstrated good internal consistency reliability, with a Cronbach’s alpha of 0.773.

### Patients’ KAP towards peri-radiotherapy nutrition

**Figure 2:**
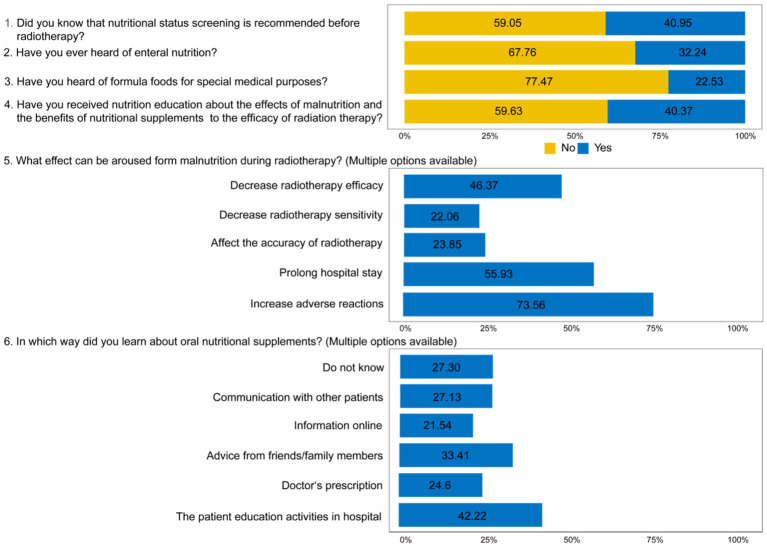
The results of knowledge questionnaire. Data represent patients’ understanding of oral nutritional supplements (ONS) and malnutrition. The knowledge section comprised 6 questions (4 single-choice and 2 multiple-choice), assessing awareness of ONS benefits, indications, and signs of malnutrition. Values are presented as the percentage of participants with the response (for single-choice items) or the percentage selecting each key option (for multiple-choice items) (*n* = 1,737).

Regarding the patients’ knowledge of nutrition, only 40.95% of the patients knew that nutrition screening is recommended before radiotherapy. Only 32.24% of patients have heard of enteral nutrition preparations, and only 22.53% of patients have heard of formula foods for special medical purposes. Only 40.37% of the patients received nutrition education about the side effects of malnutrition and the nutritional supplements that are helpful to the curative effect of radiotherapy. In terms of patients’ understanding of the possible adverse effects of malnutrition during radiotherapy, the most frequent response was “increasing adverse reactions” (73.56%), followed by “prolonging hospital stay” (55.93%, 1106/1737), and “reducing radiotherapy efficacy” (46.37%). In terms of ways for patients to know about ONS, 42.22% of patients received their information from the patient education activities in hospital and 24.6% from Doctor’s Prescription (Figure 2).

**Figure 3:**
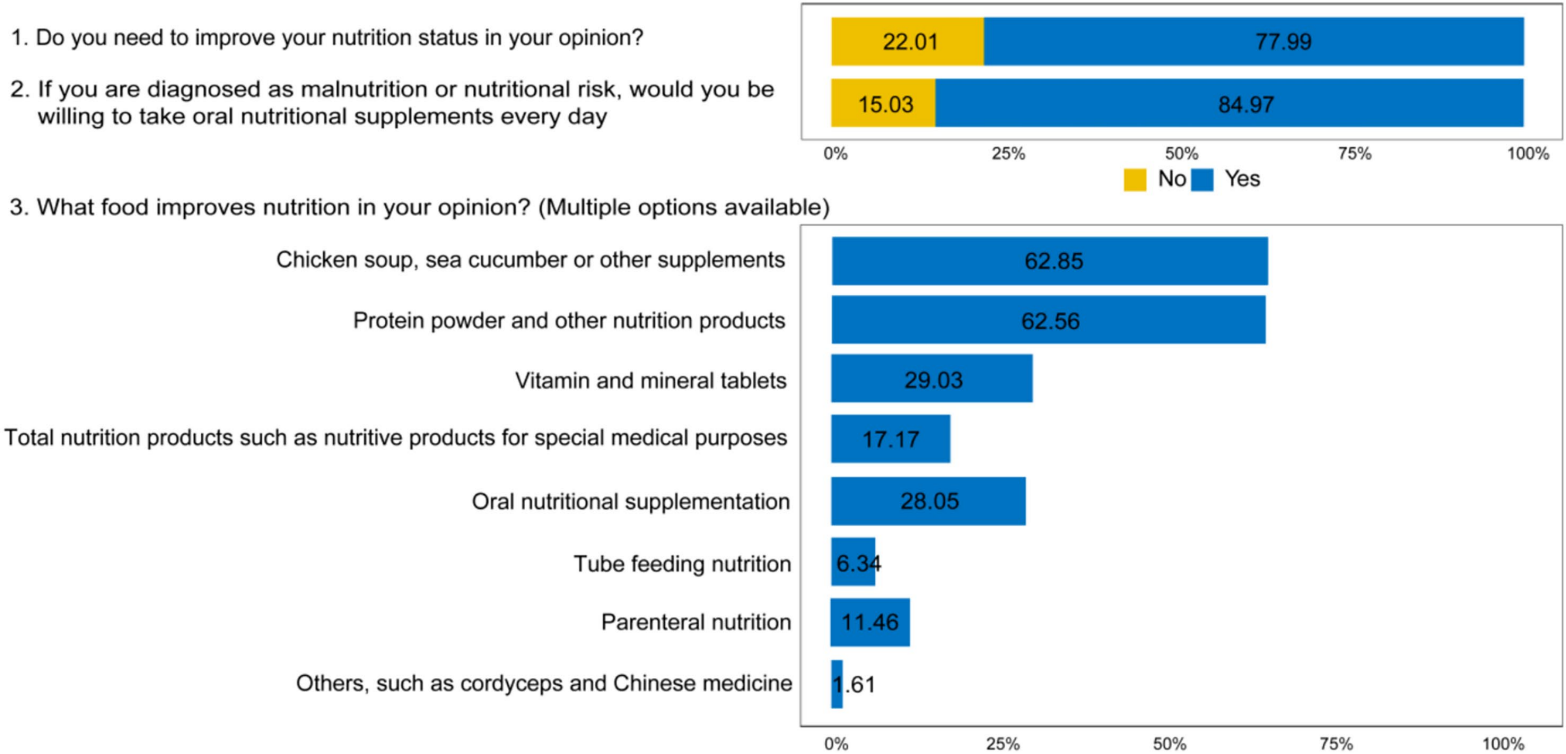
The results of attitude questionnaire. Data reflect participants’ attitudes and concerns regarding nutritional improvement during the peri-radiotherapy period. The attitude section assessed participants’ perspectives using 3 questions (2 single-choice and 1 multiple-choice), focusing on perceived importance, main concerns, and potential barriers. Values are presented as the percentage of participants selecting each given response (*n* = 1,737).

Regarding the patients’ attitudes towards nutrition knowledge during radiotherapy, the majority (77.99%) believed that nutrition should be enhanced during radiotherapy. In the case of being diagnosed with malnutrition or nutritional risks by doctors, 84.97% of the patients were willing to take ONS every day. Regarding what can effectively improve nutrition, 34.39% of the patients used the correct method of nutrition improvements such as ONS and tube feeding nutrition, while majority of patients agreed with wrong idea that Chicken soup, Sea cucumber, Cordyceps and Chinese medicine were helpful for nutrition improvement (Figure 3).

**Figure 4:**
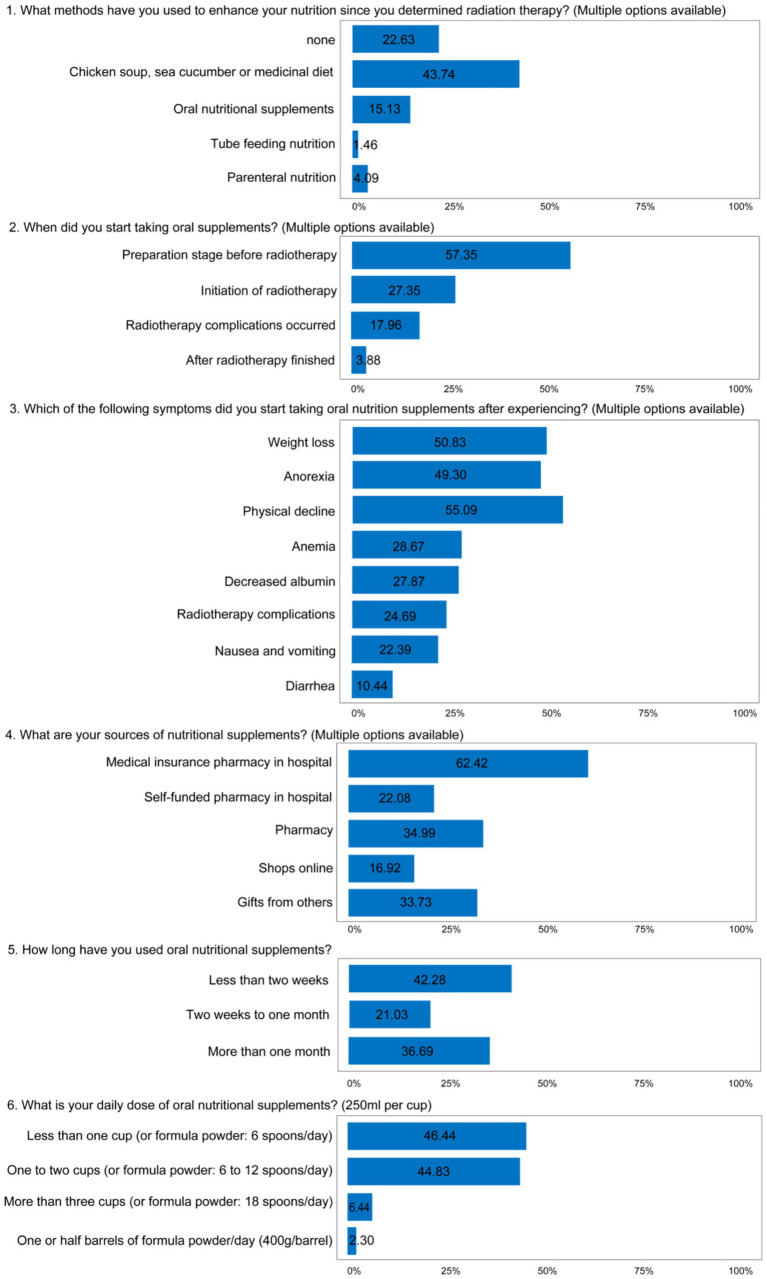
The results of practice questionnaire. Data depict patient-reported practices regarding oral nutritional supplement (ONS) usage during the peri-radiotherapy period. The practice section evaluated key aspects of ONS administration, including timing of initiation, specific methods, duration of use, and dosage. The evaluation comprised 6 questions (4 single-choice and 2 multiple-choice). Values are presented as the percentage of participants selecting each response option (*n* = 1,737).

Regarding the patients’ behavior to enhance nutrition, only 15.13% of patients took oral nutritional supplements and 1.46% received tube feeding nutrition, 4.09% patients received parenteral nutrition. In terms of the time point of taking ONS, 57.35% of the respondents started taking oral supplements during preparation stage before radiotherapy and 27.35% in the initiation stage of radiotherapy. 17.96% of patients started taking oral supplements after radiotherapy complications occurred mainly after weight loss, anorexia and physical decline occurs. 62.42% of the patients chose to obtain nutrition supplements from the hospital’s medical insurance pharmacy; 36.69% of patients were willing to follow the doctor’s advice and continue to take nutritional supplements more than 1 month. The majority of patients took less than two cups of nutritional supplements daily (Figure 4).

### Association between clinical variables and KAP score

As shown in Table 2, we listed the mean and SD values of KAP score in different groups and T test or one-way ANOVA shown that education level was associated with K score (*p* < 0.001), P score (*p* = 0.025) and KAP score (*p* < 0.001). Radiotherapy stage was associated with K score (*p* = 0.002). Therapy type (*p* < 0.001) and cancer site (*p* = 0.001) were also associated with KAP score. Afterwards, we did multiple logistic analysis to determine the significant factors for KAP score. We found that the education level was positively associated with KAP scores, suggesting that socioeconomic factors may influence nutritional awareness and behaviors (Table 3).

**Table 2:** The correlation between clinical variables and KAP score.

Variables	Classification	Knowledge score		Attitude score		Practice score		KAP score	
		Mean ± SD	*p* value	Mean ± SD	*p* value	Mean ± SD	*p* value	Mean ± SD	*p* value
Gender	Male	6.94 ± 3.01	0.477	12.08 ± 6.04	0.043*	8.16 ± 10.05	0.200	27.13 ± 14.16	0.063
	Female	6.83 ± 3.07		11.51 ± 5.51		7.56 ± 9.50		22.79 ± 10.61	
Age	<70 years	6.97 ± 3.01	0.030*	11.83 ± 5.79	0.841	7.84 ± 9.71	0.644	28.36 ± 14.66	0.821
	≥70 years	6.56 ± 2.95		11.76 ± 5.91		8.12 ± 10.26		31.46 ± 14.66	
Education level	No education	5.47 ± 2.96	<0.001***	10.17 ± 5.91	0.059	5.65 ± 8.79	0.025*	21.29 ± 14.05	<0.001***
	Informal education	6.55 ± 2.94		11.61 ± 5.78		7.11 ± 9.31		25.27 ± 13.81	
	Primary education	7.11 ± 3.05		12.20 ± 6.02		8.57 ± 9.99		27.87 ± 14.52	
	Secondary education	7.19 ± 3.07		11.73 ± 5.68		8.18 ± 9.69		27.10 ± 14.07	
	Tertiary education	7.25 ± 3.07		12.18 ± 5.43		8.63 ± 11.19		28.06 ± 15.62	
Radiotherapy stage	Before	7.03 ± 2.95	0.002**	11.72 ± 6.28	0.065	8.52 ± 11.15	0.509	31.87 ± 16.37	0.099
	Middle	6.69 ± 2.97		11.60 ± 5.57		7.71 ± 9.48		27.67 ± 14.24	
	After	7.28 ± 3.04		12.35 ± 6.09		7.98 ± 9.83		26.60 ± 14.35	
Clinical stage	I	7.33 ± 3.31	0.062	11.81 ± 5.68	0.091	8.28 ± 10.73	0.002**	27.43 ± 14.55	0.001**
	II	6.92 ± 3.08		11.66 ± 5.91		7.05 ± 9.23		25.63 ± 13.27	
	III	6.69 ± 2.89		12.1 ± 5.97		7.6 ± 9.29		26.39 ± 13.51	
	IV	7.22 ± 3.05		12.16 ± 5.93		9.21 ± 10.93		28.59 ± 15.73	
Therapy	Radiotherapy only	6.74 ± 2.99	<0.001***	11.73 ± 5.72	<0.001***	7.83 ± 9.72	0.017*	26.30 ± 14.28	<0.001***
	Concurrent chemo-radiotherapy	7.32 ± 3.29		12.60 ± 6.19		8.97 ± 10.31		28.89 ± 15.05	
	Sequential chemo-radiotherapy	7.03 ± 2.80		11.85 ± 5.78		6.69 ± 8.81		25.56 ± 12.80	
Cancer type	Head and neck cancer	7.85 ± 3.50	0.556	11.72 ± 5.52	<0.001***	7.97 ± 9.70	0.015*	27.54 ± 13.99	0.001**
	Thoracic cancer	7.59 ± 3.21		11.43 ± 5.74		7.06 ± 9.18		26.07 ± 13.35	
	Gastrointestinal cancer	7.83 ± 3.29		12.65 ± 6.00		8.86 ± 10.33		29.34 ± 14.58	
	Others	7.70 ± 3.19		11.60 ± 5.80		8.32 ± 10.34		27.62 ± 14.51	

*p < 0.05; **p < 0.01; ***p < 0.001.

**Table 3:** Association between clinical variables and malnutrition risk.

Variables	Classification	Malnutrition risk	*p* value
Age	<70 years	Ref	
	≥70 years	10.89 (8.23, 14.41)	<0.001***
Gender	Male	Ref	
	Female	0.60 (0.49, 0.74)	<0.001***
Education level	No education	Ref	
	Informal education	0.46 (0.28, 0.78)	0.003**
	Primary education	0.31 (0.18, 0.52)	<0.001***
	Secondary education	0.34 (0.20, 0.58)	<0.001***
	Tertiary education	0.26 (0.14, 0.47)	<0.001***
Radiotherapy stage	Before	Ref	
	Middle	0.91 (0.67, 1.25)	
	After	0.97 (0.69, 1.37)	
Clinical stage	I	Ref	
	II	1.19 (0.67, 2.13)	0.557
	III	1.38 (0.80, 2.38)	0.242
	IV	1.44 (0.84, 2.45)	0.186
Therapy	Radiotherapy only	Ref	
	Concurrent chemo-radiotherapy	0.91 (0.71, 1.18)	0.480
	Sequential chemo-radiotherapy	0.64 (0.47, 0.86)	0.004**
Cancer type	Head and Neck cancer	Ref	
	Thoracic cancer	1.16 (0.80, 1.69)	0.421
	Gastrointestinal cancer	2.06 (1.41, 3.02)	<0.001***
	Others	1.35 (0.90, 2.03)	0.149

*p < 0.05; **p < 0.01; ***p < 0.001.

### Association between clinical variables and malnutrition risk

Among the patients, 519 (29.9%) were identified as being at risk of malnutrition. The associations between clinical variables and malnutrition risk were assessed using the chi-square test. We found that gender (*p* < 0.001), age (*p* < 0.001), education level (*p* < 0.001), and therapy type (*p* = 0.020) were significantly associated with malnutrition risk. The other variables, such as radiotherapy stage, clinical stage and cancer type were not significant (Table 4).

**Table 4:** Association between clinical variables and malnutrition risk.

Variables	Classification	Malnutrition		*p* value
		Yes	No	
Gender	Male	623	330	<0.001***
	Female	595	189	
Age	<70 years	1,132	285	<0.001***
	≥70 years	86	234	
Education level	No education	31	35	<0.001***
	Informal education	383	201	
	Primary education	399	140	
	Secondary education	270	104	
	Tertiary education	135	39	
Radiotherapy stage	Before	159	72	0.795
	Middle	734	304	
	After	325	143	
Clinical stage	I	61	20	0.419
	II	164	64	
	III	302	137	
	IV	376	177	
Therapy	Radiotherapy only	495	242	0.020*
	Concurrent chemoradiotherapy	307	137	
	Sequential chemoradiotherapy	235	73	
Cancer type	Head and neck cancer	177	58	0.226
	Thoracic cancer	675	307	
	Gastrointestinal cancer	171	74	
	Genitourinary cancer	120	47	
	Hematologic cancer	16	12	
	Others	59	21	

*p < 0.05; **p < 0.01; ***p < 0.001.

### Association between KAP score and malnutrition risk

Finally, we conducted logistic analysis of KAP score predicting malnutrition risk. Before adjusting for clinical variables, higher K, A, P, KAP score were associated with lower malnutrition risk. After multivariate adjustment for clinical variables, such as age, gender, education, clinical stage, therapy type, radiotherapy stage and diagnosis, we also found the prediction value of higher K, A, P, KAP score for lower malnutrition risk (Table 5). Furthermore, we conducted subgroup analyses, and the results remained robust (Table 6).

**Table 5:** Association between KAP score and malnutrition risk.

Variables	Non-adjusted		Multivariate adjusted^*^	
	OR (95% CI)	*p*-value	OR (95% CI)	*p*-value
K score, quartile				
Q1	Ref		Ref	
Q2	0.88 (0.64, 1.20)	0.425	0.84 (0.60, 1.18)	0.320
Q3	0.72 (0.54, 0.97)	0.029*	0.70 (0.51, 0.95)	0.024*
Q4	0.53 (0.39, 0.73)	<0.001***	0.53 (0.38, 0.73)	<0.001***
A score, quartile				
Q1	Ref		Ref	
Q2	0.89 (0.67, 1.19)	0.435	0.90 (0.66, 1.21)	0.478
Q3	0.72 (0.54, 0.97)	0.028*	0.69 (0.51, 0.94)	0.019*
Q4	0.60 (0.45, 0.81)	<0.001***	0.55 (0.40, 0.76)	<0.001***
P score, quartile				
Q1	Ref		Ref	
Q2	0.99 (0.72, 1.35)	0.927	1.00 (0.72, 1.40)	0.999
Q3	0.93 (0.70, 1.23)	0.605	0.89 (0.66, 1.20)	0.433
Q4	0.69 (0.51, 0.94)	0.018*	0.67 (0.48, 0.93)	0.017*
KAP score, quartile				
Q1	Ref		Ref	
Q2	0.80 (0.60, 1.05)	0.110	0.74 (0.55, 1.00)	0.047*
Q3	0.47 (0.35, 0.63)	<0.001***	0.42 (0.30, 0.58)	<0.001***
Q4	0.53 (0.39, 0.71)	<0.001***	0.48 (0.35, 0.66)	<0.001***

*p < 0.05; **p < 0.01; ***p < 0.001.

**Table 6:** Subgroup analysis for the association between KAP score and malnutrition risk.

Variables	Classification	KAP score			
		Q1	Q2	Q3	Q4
Age	<60 years	Ref	0.45 (0.27, 0.74)	0.21 (0.12, 0.38)	0.42 (0.25, 0.70)
	≥60 years	Ref	0.97 (0.66, 1.41)	0.60 (0.40, 0.90)	0.56 (0.37, 0.84)
Gender	Male	Ref	0.71 (0.47, 1.08)	0.38 (0.25, 0.59)	0.46 (0.30, 0.70)
	Female	Ref	0.69 (0.42, 1.11)	0.40 (0.23, 0.69)	0.41 (0.24, 0.70)
Education level	No and informal education	Ref	0.63 (0.39, 1.02)	0.40 (0.23, 0.69)	0.37 (0.22, 0.65)
	Primary, secondary, and tertiary education	Ref	0.75 (0.50, 1.14)	0.37 (0.24, 0.58)	0.47 (0.31, 0.72)
Radiotherapy stage	Before and middle	Ref	0.72 (0.50, 1.03)	0.38 (0.26, 0.56)	0.40 (0.27, 0.60)
	After	Ref	0.65 (0.34, 1.24)	0.41 (0.21, 0.81)	0.55 (0.29, 1.06)
Clinical stage	I and II stage	Ref	0.75 (0.34, 1.63)	0.38 (0.15, 0.91)	0.48 (0.21, 1.12)
	III and IV stage	Ref	0.68 (0.45, 1.04)	0.35 (0.22, 0.55)	0.43 (0.27, 0.66)
Therapy	Radiotherapy only	Ref	0.82 (0.51, 1.32)	0.39 (0.23, 0.64)	0.48 (0.28, 0.82)
	Chemo-radiotherapy	Ref	0.70 (0.42, 1.16)	0.54 (0.32, 0.90)	0.56 (0.34, 0.93)
Cancer type	Head and neck cancer	Ref	0.62 (0.21, 1.87)	0.36 (0.11, 1.16)	0.87 (0.29, 2.62)
	Thoracic cancer	Ref	0.77 (0.49, 1.24)	0.36 (0.21, 0.63)	0.36 (0.20, 0.62)
	Gastrointestinal cancer	Ref	0.48 (0.25, 0.92)	0.48 (0.25, 0.93)	0.41 (0.22, 0.79)
	Others	Ref	0.76 (0.36, 1.61)	0.25 (0.11, 0.57)	0.36 (0.17, 0.79)

## Discussion

Cancer patients have a particularly high risk of malnutrition. ONS plays a critical role among radiation oncologists and patients to improve the clinical outcomes of radiotherapy. This study aimed to assess the KAP towards ONS during the peri-radiotherapy period among patients in Shandong Province, China. In addition, the potential relationship between malnutrition risk and KAP on ONS during the peri-radiotherapy period was examined. The results suggest that the proportion of patients receiving professional nutrition education is low. Patients with lower education level had a lower KAP score. The logistic analysis revealed that KAP score was negatively associated with malnutrition risk. Patients with higher KAP score would suffer less malnutrition risk.

Regarding patients’ knowledge of nutrition during radiotherapy, most were able to accurately recognize the negative impact of malnutrition on treatment outcomes. However, fewer than half were aware that nutrition screening or education for malnutrition is recommended before starting radiotherapy. Despite their understanding of the detrimental effects of malnutrition, the majority of patients expressed a strong need for improved nutritional support. On the other hand, many patients held misconceptions about effective methods to enhance their nutritional status. Approximately two-thirds were familiar with traditional supplements such as sea cucumber, protein powder, chicken soup, ginseng, and others, while one-third knew about *Cordyceps sinensis* and *Ganoderma lucidum*. Less than 30% were aware of oral nutritional supplements (ONS) or tube feeding as viable nutritional interventions. These findings underscore the need to improve patient education on proper nutritional support. The observed association between higher KAP scores and reduced malnutrition risk may be attributed to increased patient awareness, earlier initiation of nutritional support, and better adherence to ONS recommendations.

These findings emphasize the critical need for integrating structured nutritional education into routine oncology care. Targeted interventions aimed at improving KAP could provide a scalable and cost-effective approach to reducing malnutrition risk among cancer patients. In clinical practice, it has been observed that patient education significantly enhances both nutritional knowledge and perceptions, as well as related behaviors. In recent years, considerable efforts have been made in the field of oncology nutrition in China, including increasing the nutritional awareness of oncologists, offering training courses, organizing various competitions, evaluating standardized nutritional treatment wards for cancer radiotherapy, and establishing oncology nutrition demonstration centers. These initiatives have improved the knowledge, attitudes, and practices (KAP) of medical and nursing staff, which in turn has benefited patients. Furthermore, oral nutritional supplements (ONS) have been incorporated into China’s national medical insurance system.

ONS is the preferred nutritional therapy for cancer patients. ONS increases the intake of energy and protein, and provides balanced nutrition for patients to meet the needs of the body for nutrients (16). In this study, we investigated the access through which patients acquired nutrition knowledge. Among the patients, 42.2% obtained nutrition knowledge through hospital education, 33.41% through friends or family suggestions, 27.13% through communication with patients, 24.6% from doctors’ prescriptions, and 21.54% from online website. Therefore, we found that a large proportion of patients cannot get professional advice through doctors, nurses or hospital education channels, but through personal access. The information from the personal access is unprofessional and inaccurate. As shown in Table 3, we found that education level of tumor patients was closely related to KAP score. Still, the knowledge varied greatly regarding the components of ONS, again highlighting the need for education. As reported in previous publications, many patients did not consider nutritional intervention to be a necessary component of treatment (16). The follow-up of colorectal cancer patients for an average of 6.5 years showed improved survival for those who received nutritional counseling during radiation therapy (31).

The findings of this study align with existing epidemiological evidence on the risk of malnutrition among cancer patients undergoing treatment. For example, a recent study conducted at an oncology outpatient clinic in Spain, which assessed nearly 600 patients with locally advanced or metastatic solid tumors receiving chemotherapy, radiotherapy, and immunotherapy, similarly reported a high prevalence of malnutrition risk (32). This investigation highlighted the importance of systematic nutritional assessment in radiotherapy settings and identified several associated factors, including comorbidity indices. Our results, which demonstrate a significant association between lower KAP scores and higher malnutrition risk in peri-radiotherapy patients, complement and extend these findings by emphasizing the role of patient-level knowledge, attitudes, and practices. Future research could integrate clinical variables, such as comorbidity indices, to provide a more comprehensive understanding of the multifactorial determinants of malnutrition during cancer therapy, thereby guiding more targeted nutritional interventions.

Therefore, it is crucial for medical institutions to enhance nutritional education for patients, encouraging them to acquire accurate nutrition knowledge and proper nutritional management strategies during the peri-radiotherapy period.

In terms of KAP score, multivariable logistic analysis revealed that education level was positively associated with K, P and KAP score. People received higher education may have good economic status and are more accessible for nutrition knowledge from book or online. Moreover, they might more readily comprehend the importance of nutritional supplementation and appropriate methods for its use. Popularization of education is conducive to improving the level of patients’ cognition for nutrition supplements. Besides, in the univariable analysis, we also found that TNM stage were associated with KAP score and patients with TNM stage IV had highest KAP score. We also found that therapy type was also significantly associated with KAP score. The patients received concurrent chemoradiotherapy got the highest score. We speculated that patients with late stage and receiving concurrent chemoradiotherapy have more eating problems and longer course of disease. They may accept more nutrition intervention. In the further multivariable analysis, TNM stage and therapy type were not significant variables.

The representativeness of the surveyed population supports the applicability of the findings to similar populations in other countries. Patients’ malnutrition risks are universal. The results of the present study are supported by previous studies. Asadi et al. (33) showed a low KAP of nutrition among breast cancer patients. Other study also revealed that nutrition is often overlooked in cancer therapy (34). The univariable and multivariable logistic regression analyses on related factors of nutritional status of tumor patients during the peri-radiotherapy were performed. It is shown that education level was significant confers a nutritional risk (35).

This study is a multicenter, large-scale clinical investigation in Shandong Province designed to comprehensively assess the knowledge, attitudes, and practices regarding oral nutritional supplements among cancer patients.

This study has several limitations. First, its cross-sectional design precludes causal inference. Second, self-reported KAP data may introduce reporting bias. Third, the study was conducted in a single province, which may limit generalizability. Future prospective and interventional studies are warranted to validate these findings.

## Conclusion

Higher KAP toward ONS is significantly associated with reduced malnutrition risk in cancer patients during the peri-radiotherapy period. Enhancing patient education and implementing standardized nutritional management strategies may improve clinical outcomes.

## Data Availability

The original contributions presented in the study are included in the article/supplementary material, further inquiries can be directed to the corresponding author.
