# Supplementary material for: The knowledge, attitude, and practice score on oral nutrition supplementation is inversely associated with malnutrition risk of tumor patients during the peri-radiotherapy period: a multicenter cross-sectional study
Source: Front Nutr. 2026 Apr 29;13:1754291. doi: 10.3389/fnut.2026.1754291 (PMC13170369; doi:10.3389/fnut.2026.1754291)
Supplement: Supplementary file 1 [file Supplementary_file_1.docx]

**Oral Nutrition Supplementation** **Survey During the Peri-radiotherapy Period of Patients in Shandong province, China**

Patient Name: ________________

Hospital Name: ________________

Date of Completion: ________________

Instructions for Completion

1. The hospitalization number refers to the number assigned during your current hospital stay. If you have been discharged, please provide the number from your most recent stay.
2. For all sections with options, please circle your selected option and then write the number of your choice in the "Confirmation Column," like "[ ]".
3. Shaded areas are mandatory.
4. After completing parts one to three, please return this form to the survey staff.
5. In this survey, "oral nutritional supplementation" is defined as the use of commercial enteral nutrition formulations for oral supplementation.

**Patient Basic Information**

Hospital Name: _______________________

Patient Name: ____________ Hospitalization Number: ____________

Gender: Male Female

Date of Birth: __________ Age: _____________

Contact Mobile Number:__________________

Education Level: _______________________

Radiotherapy Stage: Pre-radiotherapy preparation During radiotherapy Within three months after completing all radiotherapy

Start Date of Radiotherapy: ____________________

End Date of Radiotherapy: ____________________

Is this the first time undergoing radiotherapy? Yes No____

**Part A: Knowledge on Nutrition** **during the Peri-radiotherapy period**

For questions and options in Part A, please circle the option that matches your situation in [ ].

A-1 Do you know your current weight? [ ]

0 No 1 Yes

A-2 Did you know that nutritional status screening is recommended before radiotherapy?[ ]

0 No 1 Yes

A-3 Have you ever heard of enteral nutrition?[ ]

0 No 1 Yes

A-4 Have you heard of formula foods for special medical purposes?[ ]

0 No 1 Yes

A-5 "Properly enhancing nutrition can help improve nutritional status, maintain treatment continuity, and improve prognosis," How do you think?[ ]

0 Incorrect 1 Correct 9 Don't know

A-6 Have you received nutrition education about the effects of malnutrition and the benefits of

nutritional supplements to the efficacy of radiation therapy (such as, lecture, videos and promotional materials)?[ ]

0 No 1 Yes

A-7 What effect can be aroused form malnutrition during radiotherapy? (Multiple options available )[ ]

(1) Increase adverse reactions, (2) Prolong hospital stay (3) Affect the accuracy of radiotherapy

(4) Decrease radiotherapy sensitivity (5) Decrease radiotherapy efficacy

A-8 In which way did you learn about oral nutritional supplements? (Multiple options available)[ ]

(1) The patient education activities in hospital (2) Doctor's prescription (3) Advice from friends/family members (4) Information online (5) Communication with other patients 6 do not know

**Part B: Attitude Towards Nutritional Supplementation during the Peri-radiotherapy period**

For questions and options in Part B, please circle the option that matches your situation in [ ].

B-1 Do you think you need to enhance your nutrition?[ ]

0 No 1 Yes

B-2 What food improves nutrition in your opinion? (Multiple options available)[ ]

1. Chicken soup, sea cucumber or other supplements
2. Protein powder and other nutrition products
3. Vitamin and mineral tablets
4. Total nutrition products such as nutritive products for special medical purposes
5. Oral nutritional supplementation
6. Tube feeding nutrition
7. Parenteral nutrition
8. Others, such as cordyceps and Chinese medicine

B-3 If diagnosed with malnutrition or at risk of malnutrition, which treatment recommendation are you willing to follow?[ ]

1. Nutritional drugs covered by health insurance
2. Nutritional products at your own expense from the hospital pharmacy
3. Nutritional powder made by the hospital's nutrition department
4. Nutritional products from supermarkets

B-4 If you are diagnosed as malnutrition or nutritional risk, would you be willing to take oral nutritional supplements every day (such as healthcare supplements)?[ ]

1 yes, 0 no

**Part C: Nutritional Supplementation Behavior during the Peri-radiotherapy period**

For questions and options in Part C, please circle the option that matches your actual situation in [ ]

C-1 What methods have you used to enhance your nutrition since you determined radiation therapy?

1. none
2. Chicken soup, sea cucumber or medicinal diet
3. Oral nutritional supplements (such as total nutrition powder, protein powder, vitamin and mineral tablets)
4. Tube feeding nutrition
5. Parenteral nutrition

If option (3) is not selected, your questionnaire is complete;

If option (3) is selected, please continue with the questions in this part.

C-2 When did you start oral nutritional supplementation? [ ]

1. Before radiotherapy preparation
2. At the start of radiotherapy
3. When complications from radiotherapy occurred (difficulty swallowing, poor appetite, etc.)
4. Within three months after completing all radiotherapy

c-3 Which of the following symptoms did you start taking oral nutrition supplements after experiencing them (multiple options available)? [ ]

1. Weight loss
2. Anorexia
3. Physical decline
4. Anemia
5. Decreased albumin
6. Radiotherapy complications (dysphagia, mucositis, etc)
7. Nausea and vomiting
8. Diarrhea

C-4 What are your sources of nutritional supplements? (Multiple options available)[ ]

1. Medical insurance pharmacy in hospital
2. Self-funded pharmacy in hospital
3. Pharmacy
4. Shops online
5. Gifts from others
6. Others

C-5 How long have you used oral nutritional supplements?[ ]

1. Less than two weeks
2. Two weeks to one month
3. More than one month

C-6 When will you stop taking oral nutritional supplements?[ ]

1. Stop when the existing formula is used up
2. Stop after radiotherapy
3. Continue for one month after radiotherapy
4. Continue for 2-3 months after radiotherapy
5. After appetite/weight return to normal
6. According to your doctor's advice
7. Improvement of complications
8. Stopped already

C-7 What is your daily dose of oral nutritional supplements? (250ml per cup)[ ]

1. Less than one cup (or Formula powder: 6 spoons/day)
2. One to two cups (or formula powder: 6 to 12 spoons/day)
3. More than three cups (or Formula powder: 18 spoons/day)
4. One or half barrels of formula powder/day (400g/barrel)

C-8 What are the common issues you encounter with oral nutritional supplementation products and the questions you hope to get answered by medical staff? (multiple choices allowed)[ ]

1. Don't know how to use it effectively (e.g., dosage and method of intake)
2. Don't know the method of preparation (e.g., concentration)
3. Don't know the contraindications of use (e.g., can it be added to other foods? Temperature?)
4. Don't know why the doctor prescribed this formula
5. Don't know if the formula is effective
6. Don't know how long to use it?
7. What to do if the taste is not acceptable?
8. Other, please specify: _______________________________________________

**Thank you for your participation, the questionnaire is now complete!**

**Please return the questionnaire to the medical staff.**

***************************[For Medical Personnel Only]******************************

**Part D: Screening and Assessment of Nutritional Risk and Malnutrition during the Peri-radiotherapy period**

Attending/Primary Physician: ______________________________

Primary Diagnosis: ________________________________________

Secondary Diagnosis: ________________________________________

Pathological Type: ________________________________________

Staging: TNM Staging: T ____ N____ M____ (Fill in the numbers) Clinical Stage: ___

Radiotherapy Stage: Pre-radiotherapy preparation During radiotherapy Within three months after completing all radiotherapy

Start Date of Radiotherapy: _________________________

End Date of Radiotherapy: _________________________

For questions and options in Part D, please circle the option that matches your actual situation in the "Confirmation Column"

D-1 Since deciding to undergo radiotherapy, has the patient undergone nutritional screening/assessment? [ ]

(1) Yes (Continue to question D-2) (0) No (Skip to question D-5)

(3) Unsure (Skip to question D-5)

D-2 When was the patient's most recent nutritional assessment?[ ]

1. Pre-radiotherapy preparation (2) During radiotherapy (3) After completing all radiotherapy

D-3 Methods and results of the most recent nutritional assessment (multiple choices allowed)?[ ]

1. Physical measurement
2. Questionnaire assessment
3. Nutritional related blood biochemical indicators
4. Other __________________________

D-4 Conclusion of hospital nutritional status assessment: [ ]

(0) No nutritional risk (1) Nutritional risk

(2) Malnutrition

D-5 Patient's nutritional indicators (most recent blood biochemistry results or nutritional risk assessment, within a week before the date of filling out this form):

Weight: ______kg (measured at surveyed time)

Height: ______cm (measured at surveyed time)

Albumin level: _____g/L (Date: ____________)

Prealbumin level: _____ mg/L (Date: ____________)

Hemoglobin level: _____ g/L (Date: ____________)

Nutritional risk screening or nutritional status assessment questionnaire and results:

1. NRS-2002 ; Score____________(if applicable)
2. SGA；Score____________(if applicable)
3. PG-SGA；Score____________(if applicable)
4. Other: ___； Score(if applicable)
