# Supplementary material for: The knowledge, attitude, and practice score on oral nutrition supplementation is inversely associated with malnutrition risk of tumor patients during the peri-radiotherapy period: a multicenter cross-sectional study
Source: Front Nutr. 2026 Apr 29;13:1754291. doi: 10.3389/fnut.2026.1754291 (PMC13170369; doi:10.3389/fnut.2026.1754291)
Supplement: Supplementary file 2 [file Supplementary_file_2.docx]

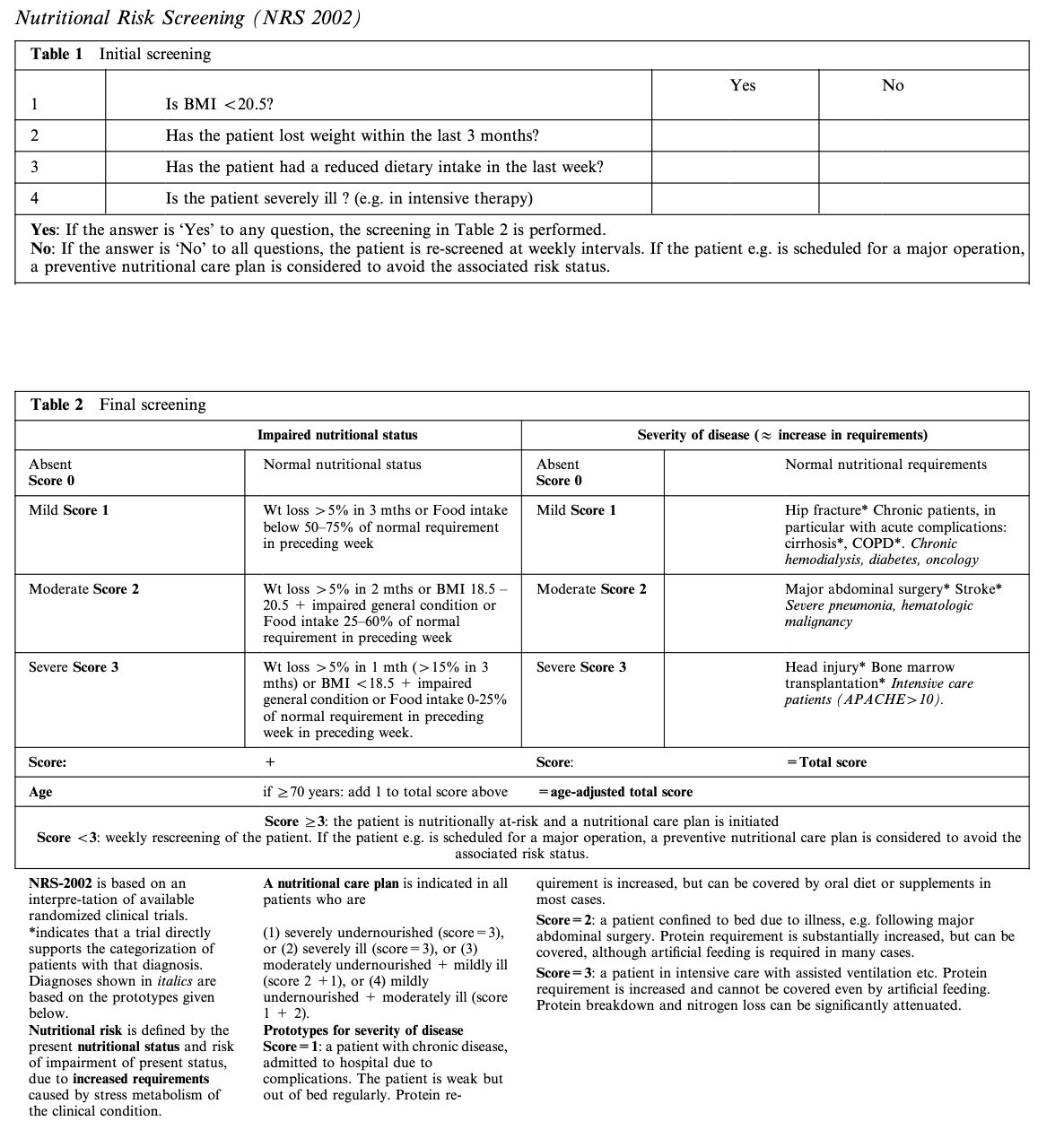
Nutritional Risk Screening (NRS 2002)

**Reference**

ESPEN guidelines for nutrition screening 2002, Clin Nutr. 2003 Aug;22(4):415-21.
